# Acute kidney injury in cats and dogs: A proportional meta-analysis of case series studies

**DOI:** 10.1371/journal.pone.0190772

**Published:** 2018-01-25

**Authors:** Sabrina Almeida Moreira Legatti, Regina El Dib, Emerson Legatti, Andresa Graciutti Botan, Samira Esteves Afonso Camargo, Arnav Agarwal, Pasqual Barretti, Antônio Carlos Paes

**Affiliations:** 1 Department of Veterinary Hygiene and Public Health, School of Veterinary Medicine and Animal Science, Unesp – Univ Estadual Paulista, Botucatu, São Paulo, Brazil; 2 Department of Anaesthesiology, Botucatu Medical School, Unesp – Univ Estadual Paulista, Botucatu, São Paulo, Brazil; 3 McMaster Institute of Urology, McMaster University, Hamilton, Ontario, Canada; 4 School of Veterinary Medicine and Animal Science, Unesp – Univ Estadual Paulista, Botucatu, São Paulo, Brazil; 5 Institute of Science and Technology, Department of Biosciences and Oral Diagnosis, Unesp – Univ Estadual Paulista, São José dos Campos, São Paulo, Brazil; 6 Schoolof Medicine, University of Toronto, Toronto, Canada; 7 Department of Internal Medicine, Botucatu Medical School, Unesp – Univ Estadual Paulista, Botucatu, São Paulo, Brazil; The University of Manchester, UNITED KINGDOM

## Abstract

**Introduction:**

Risk of mortality in the setting of acute kidney injury (AKI) in cats and dogs remains unclear.

**Objectives:**

To evaluate the incidence of mortality in cats and dogs with AKI based on etiology (i.e. infectious versus non-infectious; receiving dialysis versus conservative treatment).

**Materials and methods:**

Ovid Medline, EMBASE, and LILACS were searched up to July 2016. Articles were deemed eligible if they were case series studies evaluating the incidence of all-cause mortality in cats and dogs with AKI, regardless of etiology or the nature of treatment.

**Results:**

Eighteen case series involving 1,201animalsproved eligible. The pooled proportions for overall mortality were: cats53.1% [95% CI 0.475, 0.586; I^2^ = 11,9%, p = 0.3352]; dogs 45.0% [95% CI 0.33, 0.58; I^2^ = 91.5%, P < 0.0001]. A non-significant increase in overall mortality risk was found among dialysed animals relative to those managed with conservative treatment, independent of animal type and the etiology of their AKI. The pooled proportions for overall mortality according to etiology, regardless of treatment type, were: AKI due infectious etiology for cats and dogs, 19.2% [95% CI 0.134, 0.258; I^2^ = 37.7%, P = 0.0982]; AKI due non-infectious etiology for cats and dogs, 59.9% [95% CI 0.532, 0.663; I^2^ = 51.0%, P = 0.0211].

**Conclusion:**

Our findings suggest higher rates of overall mortality in cats and dogs with AKI due to non-infectious etiologies relative to infectious etiologies, and showed non-significant differences in terms of higher rates associated with dialysis compared to conservative management. Further investigations regarding optimal time to initiate dialysis and the development of clinical models to prognosticate the course of disease and guide optimal treatment initiation for less severe cases of AKI in cats and dogs is warranted.

## Introduction

Acute kidney injury (AKI) is defined as an abrupt decline in renal filtration characterized by elevated serum creatinine levels, acute uremia, and changes in urine volume. AKIs affect dogs and cats similar to humans, maybe associated with one or more of various contributory causes and may vary in severity[1,2]. The most commonly reported AKI in the literature are: hemodynamic decline (e.g. hypotension and hypovolemia); infectious (e.g. leptospirosis and pyelonephritis); nephrotoxic agents exposure (e.g. nonsteroidal anti-inflammatory drugs and lily poisoning); and, obstruction of the urinary tract (e.g. urolithiasis).

Conservative management of AKI involves fluid resuscitation, discontinuation and avoidance of nephrotoxic medications, nutritional support, correction of anuria or oliguria, symptom control in terms of nausea and vomiting, and correction of electrolyte and acid-base imbalances[3,4]. A number of new treatments have recently emerged for AKI management in veterinary medicine, including dialysis techniques such as hemodialysis and peritoneal dialysis. However, they are often limited to few centers internationally due to the need for special equipment and trained personnel[5,6].

Previous examination of the long-term impact of intermittent hemodialysis on cats and dogs with AKI revealed a survival rate of approximately 50%, similar to that of human patients[7]. However, to the best of our knowledge, no studies have directly compared hemodialysis to conservative management for animals with AKI.

With this in mind, our systematic review of case series studies aimed to evaluate the incidence of mortality in cats and dogs with AKI based on etiology (i.e., infectious versus non-infectious) and therapeutic strategy (i.e., receiving dialysis compared to conservative treatment).

## Materials and methods

Our reporting adheres to the Preferred Reporting Items for Systematic Reviews and Meta-analyses (PRISMA) [8] and Meta-analysis of Observational Studies in Epidemiology (MOOSE) Statements [9].

A review of clinical case series with pooled analysis of proportions of animals with AKI managed with dialysis or conservative treatment was performed. The methods for pooled analysis of case series proportions used in this study have been previously described in detail [10,11].

### Eligibility criteria

Studies were considered eligible if they met the following criteria: (i) case series studies (number of reported animals in each study greater than one), (ii), dogs and cats diagnosed with AKI, defined as rapid decrease of glomerular filtration rate with subsequent azotemia, regardless of etiology (i.e. infectious or non-infectious) (iii) reporting etiology of the AKI (infectious (leptospirosis, sepsis, pyelonephritis and pyometra) or non-infectious (nephrotoxic, obstructive, metabolic/hemodynamic, neoplastic and unknown)), (iii) AKI managed with either dialysis (hemodialysis or peritoneal dialysis) or conservative methods (fluid or fluid plus diuretics); and(iv) reporting mortality rate by time of hospital discharge, including euthanasia.

### Data source and searches

We searched the following electronic databases up to July 4 2016:US National Library of Medicine (PubMed; 1979 to July to 2016), Excerpta Medica database (EMBASE; 1979 to July to 2016) and Literatura Latino-Americana and Caribe em Ciências da Saúde (LILACS; 1979 to July to 2016). The search strategy was adapted for each database (S1 Table). The bibliographic references in relevant articles were also examined for eligible studies. No language or publication date restrictions were applied. Conference abstracts identified by the electronic search were also evaluated for eligibility.

### Selection of studies

Two reviewers independently screened all titles and abstracts identified by the literature search, obtained full-text articles of all potentially eligible studies and evaluated them for final inclusion. Reviewers resolved disagreements by discussion or, if necessary, with third party adjudication.

### Data extraction

Data extraction was conducted by paired reviewers, with discrepancies resolved by discussion. A standardized form was used to extract the following information: authors and year of publication, country, number of animals, animals’ mean age, AKI etiology, type of AKI, description of treatment and comparator groups, and outcome of interest.

Authors were contacted to clarify any missing or unclear data. Case series with incomplete data were included only in the qualitative analysis. Studies that presented other clinical condition rather than AKI (e.g. chronic renal disease) were considered to be included only whether the authors presented data on different clinical conditions separately [6,12–14].

### Statistical analysis and statistical heterogeneity

The outcomes were treated as a dichotomous variable with respective 95% confidence intervals (CI). Statistical heterogeneity was assessed with the I^2^ statistic, and significance was assumed when the I^2^ was greater than 50%. The I^2^ statistic illustrates the percentage of the variability in effect estimates resulting from heterogeneity rather than sampling error[10,11]. I^2^ helps readers to assess the consistency of the results of included studies in a meta-analysis. Assessment of the consistency of effects across studies is an essential part of meta-analysis [15]. This illustrates the percentage of the variability in effect estimates resulting from heterogeneity rather than sampling error [15,16]. I^2^ = [(Q—df)/Q] x 100% test, where Q is the chi- squared statistic and df its degrees of freedom [15,16].

We conducted sensitivity analyses, specifically excluding studies that did not include euthanasia data to test the robustness of the results. Specifically, we conducted a sensitivity analysis in which Francey 2002 study [17] was excluded due to the absence of euthanasia data.

Because of the clear differences among the included studies and several uncontrolled variables, we used a random-effect model [18] to perform a proportional meta-analysis of case series studies[10,11]. The software used to plot the studies in the meta-analysis was StatsDirect.

Forest plots with a 95% CI were generated to summarize the data. Each horizontal line on a forest plot represents a case series included in the meta-analysis. The length of the line corresponds to a 95% CI of the corresponding case series’ effect estimate. The effect estimate is marked with a solid black square. The size of the square represents the weight that the corresponding study exerts in the meta- analysis. The pooled estimate is marked with an unfilled diamond at the bottom of the forest plot. Confidence intervals of pooled estimates are displayed as a horizontal line through the diamond; this line might be contained within the diamond if the confidence interval is narrow.

Funnel plots were performed with Egger's test for each outcome in where 10 or more eligible studies were identified.

Statistically significant differences between interventions was defined as combined 95% CIs not overlapping and p<0.1.

## Results

### Study selection

**Fig 1:**
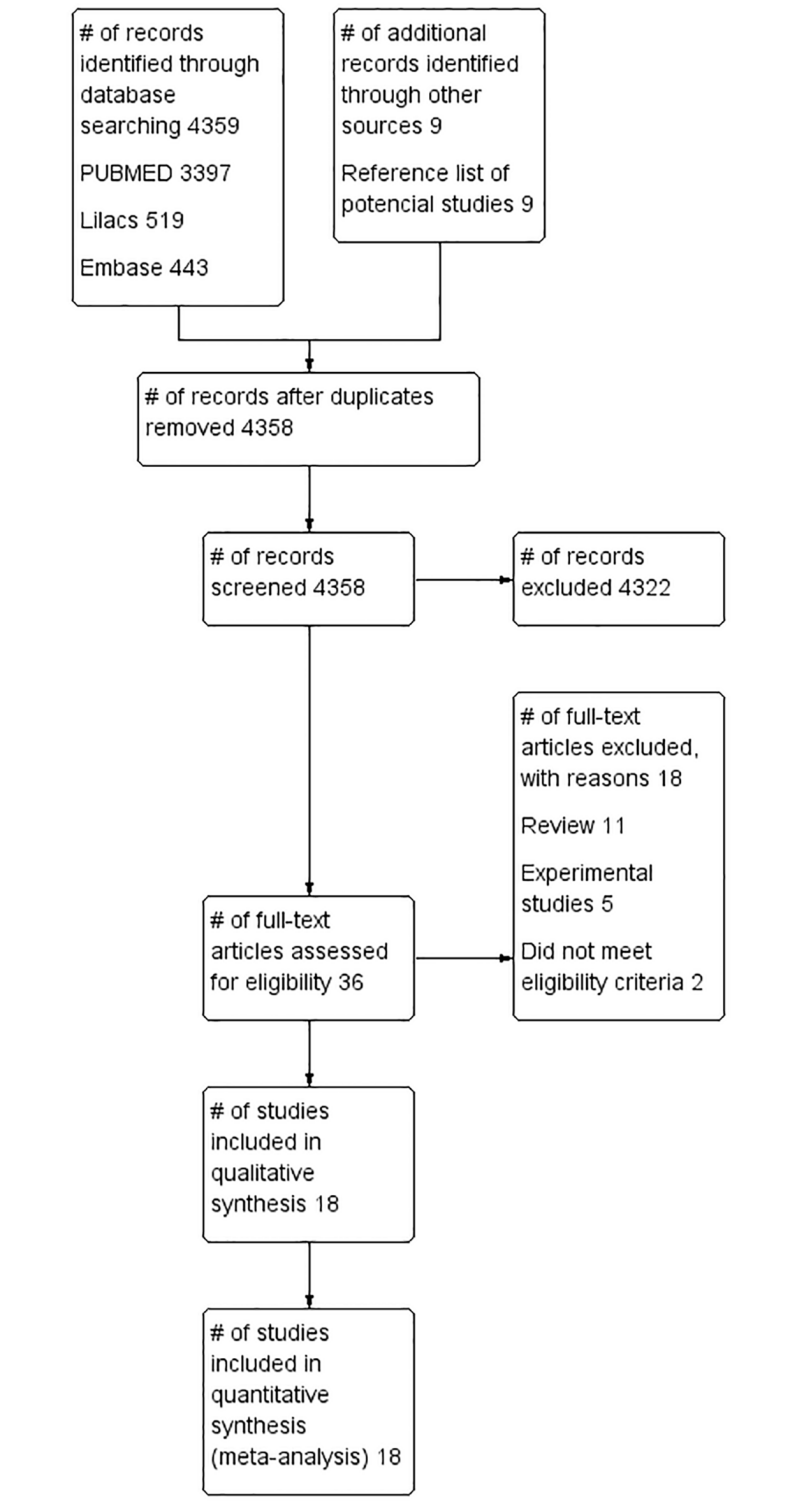
Flowchart of the review.

We identified a total of 4.358 citations after duplicates were removed. Thirty-six potentially eligible hits were identified based on title and abstract screening, of which18 case series involving 1.201 animals were deemed eligible following full-text review. The majority of the included studies (88.9%; n = 16) were available as full-text articles, while11.1% (n = 2) were conference abstracts (Fig 1).

We contacted the authors of six included studies, and five [7,14,19–21] supplied us with requested information.

### Study characteristics

Fourteen studies were conducted in the USA[1,4–6,12–14,17,19,20,22–25], two in Canada [7,26], one study[21] was conducted in Europe, and one in Central and South America [27]. Case series sample size ranged from three[21] to 182[19] animals, with a mean age of 7.29 years. The same number of studies evaluated AKI with infectious (n = 15, 353 animals) and non-infectious etiologies (n = 15, 726 animals)(Table 1).

**Table 1:** Characteristics of small animals: Comparison among different AKI treatment and etiology.

		Treatment		Etiology	
	Total	Dialytic	Conservative	Infectious	Non-infectious
**Total** #**of case series**	18	13[1,22,28]	8[1,22,28]	15^₠^	15^₠^
**Total** #**of animals**	1.201^$^	801	400	353	726
#**of male (%)**	526 (43.8)	-	-	-	-
**Mean age (years)**	7.29	-	-	-	-
**Total** #**of (%)**					
**Cats**	401 (33.4)	357	44	22	313
**Dogs**	800 (66.6)	444	356	328	394
**Type of AKI (%)**					
**Oliguric**	249(20.7)	220 (88.4)	29 (11.6)	-	-
**Non-oliguric**	304 (25.3)	142 (46.7)	162 (53.3)	-	-
**Unknown**	88 (7.3)	21 (23.9)	67 (76.1)	-	-
**Total** #**of animals (%) per etiology**					
**• Infectious**	353 (29.4)	328 (92.9)	22 (6.2)	353 (29.4)	NA
**Leptospirosis**	154 (43.6)	-	-	154 (12.8)	NA
**Pyelonephritis**	20 (5.7)	-	-	20 (1.7)	NA
**Pyometra**	132 (37.4)	-	-	132 (11.0)	NA
**Sepsis**	9 (2.5)	-	-	9 (0.7)	NA
**• Non-infectious**	726 (60.4)	394 (54.3)	313 (43.1)	NA	726
**Nephrotoxic**	220 (30.3)	-	-	NA	220 (18.3)
**Obstructive**	115 (15.8)	-	-	NA	115 (9.6)
**Unknown**	265 (36.5)	-	-	NA	265 (22.1)
**Country** **(**# **of studies)**					
**Europe**	1	1	0	0	1
**USA**	14	10	7	10	10
**Central and South America**	1	0	1	1	0
**Canada**	2	2	0	2	2
**Mean follow-up (months)**	5.86	6.5	2	5.86	5.86

^#^: Number.

NA: not applied; USA: United States of America.

^₠^Twelve articles [1,5–7,13,17,19,20,23–26] studied both types of etiologies.

^$^Number of cats and dogs from the 18 included studies.

Table 2 describes study characteristics related to description of the intervention, inclusion and exclusion criteria. All the included studies reported on a confirmed diagnosis of AKI based on the value increased creatinine.

**Table 2:** Study characteristics related to population, intervention or comparator groups, etiology of AKI, and eligibility criteria.

Author, year	#of animals	Description of intervention	Description of comparator	Etiology of AKI	Inclusion criteria	Exclusion criteria
Adin, 2000 [22]	Dogs: 36	Hemodialysis	Conservative treatment	Infectious	A single serum antibody titer ≥ 1:800 against any of 6 serovars of *L*. *interrogans* or evidence of seroconversion on paired serum samples, and clinical signs of leptospirosis.	NR
Behrend, 1996 [23]	Dogs: 29	Conservative treatment	Not applied	Infectious and non-infectious	Medical records the animals with serum creatinine ≥ 2,5 mg/ dl. Only animals that developed AKI while in a veterinary hospital or under the care of a veterinary were included in the study.	NR
Brown, 2015 [1]	Dogs: 10	Hemodialysis	Conservative treatment	Infectious and non-infectious	Inclusion criteria were acute onset of clinical signs (<7 days), serum creatinine concentration >5 mg/dL, urine production <0.5 mL/kg/h, urine specific gravity <1.025, absence of ultrasonographic evidence of chronic kidney disease, and body weight >15 kg.	NR
Cooper, 2011 [12]	Cats: 22	Peritoneal Dialysis	Not applied	Non-infectious	Criteria for inclusion in the study consisted of diagnosis of acute kidney injury and at least 1 PD cycle performed. Acute kidney injury was defined as a severe and sudden decrease in glomerular filtration rate and subsequent uremia.	Cats with uroabdomen because of bladder rupture.
Crisp, 1989 [13]	Dogs: 25 Cats: 2	Peritoneal Dialysis	Not applied	Infectious and non-infectious	Patients treated with peritoneal dialysis (January 1976—January 1987) that had serum blood creatinine > 10 mg/dl.	NR
Dorval, 2009 [26]	Cats: 6	Peritoneal Dialysis	Not applied	Infectious and non-infectious	All cats managed with PD for ARF between January 2003 and December 2007. Only cats diagnosed with ARF and a potentially reversible underlying disease were selected for PD.	Cats with signs suggestive of chronic kidney disease and cats believed to have an irreversible underlying disease were not selected for PD.
Eatroff, 2012 [7]	Dogs: 93 Cats: 42	Hemodialysis	Not applied	Infectious and non-infectious	Cats and dogs that were treated with hemodialysis intermittent at the Bobst Hospital of the Animal Medical Center between January 1997 and October 2010.	Diagnosis of CKD made prior to or during the course of treatment with IHD, treatment with IHD as a blood purification treatment for an acute intoxication, the absence of a complete medical record, the use of continuous renal replacement therapy in addition to IHD, a concurrent diagnosis of neoplasia, and renal transplantation mediatelyfollowing IHD
Ferreira, 2010 [27]	Dogs: 132	Conservative treatment	Not applied	Infectious	132 female dogs with pyometra and AKI from 22 October 2004 to 17 February 2006. Inclusion criteria: creatinine ≥ 2.4 mg / dL and/or increases of 100% creatinine diagnosis 24 hours after ovariohysterectomy.	NR
Francey, 2002 [17]	Dogs 124	Hemodialysis	Not applied	Infectious and non-infectious	Medical records of all dogs that received hemodialysis treatment for the management of acute renal failure from January 1990 to February 2001.	NR
Harkin, 1996 [14]	Dogs: 17	Conservative treatment	Not applied	Infectious	Medical records of dogs diagnosed with leptospirosis at the Oradell Animal Hospital in New Jersey and the Michigan State University Veterinary Clinical Center from 1990 through 1995.	NR
Langston, 1997 [6]	Cats: 29	Hemodialysis	Not applied	Infectious and non-infectious	Medical records the cats that were selected for dialysis based on severity of azotemia (serum creatinine concentration > 10 mg/dL; blood urea nitrogen [BUN] concentration > I50 mg/dL), persistent oliguria or anuria, uncontrolled hypervolemia, severe clinical manifestations of’ uremia, or presurgical conditioning for renal transplantation.	NR
Langston, 2002 [28]	Cats: 6	Hemodialysis	Conservative treatment	Non-infectious	Cats treated at the Animal Medical Center, New York, after lily ingestion presenting acute renal injury.	NR
Nielsen, 2015 [24]	Dogs: 58 Cats: 64	Conservative treatment	Not applied	Infectious and non-infectious	Medical records of cats and dogs admitted until august 2008 and June 2012 with AKI.	Incomplete medical records
Pantalco, 2004 [25]	Cats: 119	Hemodialysis	Not applied	Infectious and non-infectious	Medical records of all cats diagnosed acute uremia that was treated with hemodialysis between January 1993 and December 2003.	NR
Schweighauser, 2016 [21]	Dogs 3	Hemodialysis	Not applied	Non-infectious	Description of three cases with AKI due poisoning ethylene glycol.	NR
Segev, 2013 [5]	Cats: 132	Hemodialysis	Not applied	Infectious and non-infectious	Medical records the cats is acute uremia attended between jan 1993 and feb 2007. Acute uremia was defined by the following: acute onset of clinical signs, history and physical examination consistent with AKI or ureteral obstruction, azotemia (creatinine >3 mg/dL).	Cats with urinary system rupture, with urethral obstruction, or that underwent renal transplantation were excluded.
Segev, 2008 [19]	Dogs: 182	Hemodialysis	Not applied	Infectious and non-infectious	Dogs presented to the University of California, William R. Prichard Veterinary Medical Teaching Hospital (VMTH) with AKI and managed with hemodialysis.	CKD, Obstructive, euthanized within 2 weeks after inition of hemodialysis.
Vaden, 1997 [20]	Dogs: 99	Conservative treatment	Not applied	Infectious and non-infectious	Medical records of dogs presented to North Carolina State University, College of Veterinary Medicine teaching hospital from January 1985 to October 1993 were searched for the diagnoses of renal disease, renal insufficiency, or AKI. The following criteria were used to define AKI: clinical signs for fewer than 7 days, azotemia, normal or enlarge kidney size as detected by physical examination, survey radiography, or ultrasonography, and absence of clinicopathologic or radiographic data consistent with chronic renal failure.	Dogs with exclusively prerenal or postrenal azotemia were excluded.

^#^: Number.

ARF: Acute renal failure; AKI: Acute renal injury; BUN: blood urea nitrogen; CKD: Chronic kidney disease; IHD: intermittent hemodialysis; NR: not reported; PD: Peritoneal dialysis; sCr: Serum creatinine.

### Outcomes

#### Overall mortality according to animal species

The pooled proportions for overall mortality according to animal species, regardless of treatment type and AKI etiology, in the following groups were: cats from nine case series studies[5–7,12,13,24–26,28] with a total of 401 cats, 53.1% [95% CI 0.475, 0.586; I^2^ = 11.9%, P = 0.3352]; dogs from 12 case series studies [1,7,13,14,17,19–24,27] with a total of 800 dogs, 45.0% [95% CI 0.33, 0.58; I^2^ = 91.5%, P < 0.0001] (S1 Fig). While the association was non-significant for cats (p = 0.352); there was a significant association found for dogs (p< 0.0001).

The rate of overall mortality was higher for cats (53.1%) compared to dogs (45.0%), independent of treatment and AKI etiology, although there was no significant difference among the studied groups as their CIs overlapped (S1 Fig).

S2 Fig presents the results of a funnel plot of studies regarding overall mortality for dogs, suggesting potential publication bias related to treatment effect and study size.

#### Overall mortality according to treatment

The pooled proportions for overall mortality according to treatment were: dialysis from 13 case series studies[1,5–7,12,13,17,19,21,22,25,26,28]with a total of 777for cats and dogs, 52.7% [95% CI 0.469, 0.584; I^2^ = 47.1%, P = 0.0303]; conservative management (control group) from eight case series studies [1,14,20,22–24,27,28] with a total of 424for cats and dogs, 36.8% [95% CI 0.191, 0.565; I^2^ = 92.6%, P < 0.0001]; dialysis from eight case series studies [5–7,12,13,25,26,28] with a total of 333cats, 54.1% [95% CI 0.457, 0.623; I^2^ = 38.7%, P = 0.1214]; conservative management from two case series studies [24,28]with a total of 68cats, 50.5% [94.5% CI 0.361, 0.649; I^2^ = not applicable, P = 0.297]; dialysis from seven case series studies [1,7,13,17,19,21,22] with a total of 449 dogs, 51.0% [95% CI 0.43, 0.60; I^2^ = 54.2%, P = 0.0414]; conservative management from seven case series studies [1,14,20,22–24,27] with a total of 356 dogs, 37.0% [95% CI 0.18, 0.59; I^2^ = 93.1%, P < 0.0001]. (S3 Fig). Associations were significant for all except the dialysis (p = 0.1214) and conservative management (p = 0.297) for cats.

Although the rate of overall mortality was higher in dialysis groups, there was no significant difference among all studied groups as their CIs overlapped (S3 Fig).

S4 Fig presents the results of a funnel plot of studies regarding overall mortality for cats and dogs that receiving dialysis, showing potential publication bias related to treatment effect and study size.

#### Overall mortality according to AKI etiology

The pooled proportions for overall mortality according to etiology were: AKI due to infectious etiology from 11 case series studies[1,5–7,14,17,19,22,25–27] with a total of 329 cats and dogs, 19.2% [95% CI 0.134, 0.258; I^2^ = 37.7%, P = 0.0982]; AKI due to non-infectious etiology from 12 case series studies [1,5–7,12,17,19,21,23,25,26,28] with a total of 605 cats and dogs, 59.9% [95% CI 0.532, 0.663; I^2^ = 51.0%, P = 0.0211]; AKI due to infectious etiology from five case series studies [5–7,25,26] with a total of 22 cats, 30.0% [95% CI 0.13, 0.51; I^2^ = 21.5%, P = 0.2773]; AKI due to non-infectious etiology from seven case series studies [5–7,12,25,26,28] with a total of 313 cats, 53.3% [95% CI 0.478, 0.587; I^2^ = not applicable, P = 0.6025]; AKI due to infectious etiology from seven cases series studies [1,7,14,17,19,22,27] with a total of 307 dogs, 17.3% [95% CI 0.118, 0.236; I^2^ = 38.1%, P = 0.1382]; AKI due to non-infectious etiology from six cases series studies [1,7,17,19,21,23] with a total of 292 dogs, 68.0% [95% CI 0.62, 0.74; I^2^ = 12.0%, P = 0.3385](S5 Fig). The only significant associations were those of AKI due to infectious etiology (p = 0.0982) and non-infectious etiology (p = 0.0211) for both cats and dogs.

**Fig 2:**
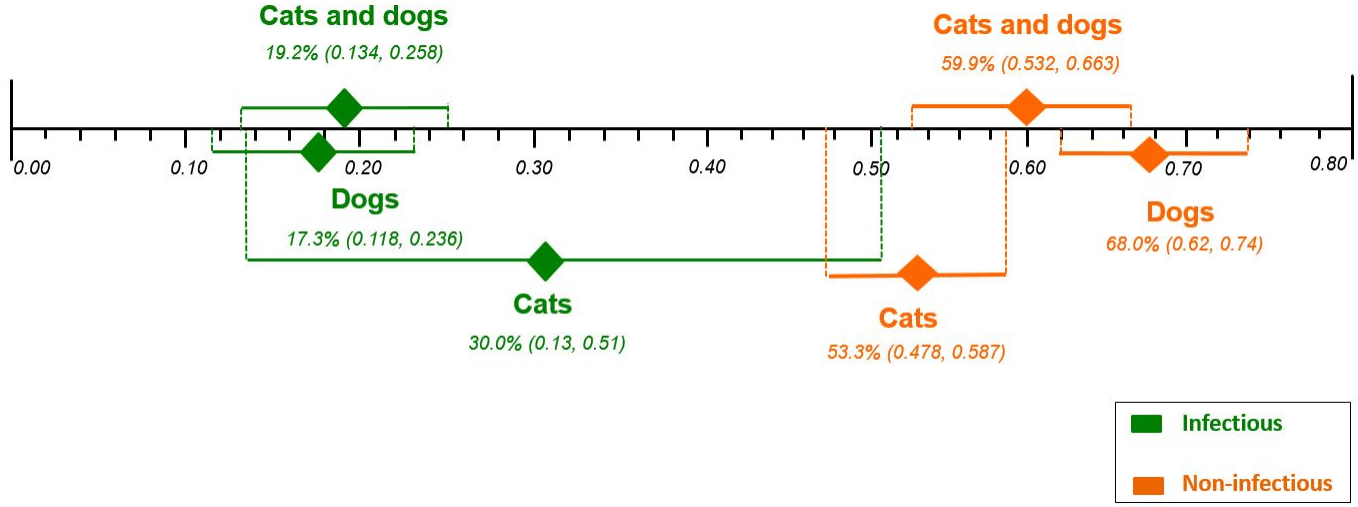
Comparison of the plotted proportional meta-analysis, according to etiology in both cats and dogs, regardless treatment, for overall mortality. Effect differences were seen due to the non-overlap of the 95% confidence intervals showing a higher rates of mortality in the AKI due to non-infectious (cats and dogs; and only dogs) compared with AKI due to infectious (cats and dogs; and only dogs), as their CIs did not overlap. However, there was no statistically significance difference between rates of mortality by etiology in only cats, as their CIs overlapped.

Effect differences were seen, showing higher rates of mortality in AKI due to non-infectious etiologies(cats and dogs, 59.9%; dogs, 68.0%) compared to AKI due to infectious etiologies (cats and dogs, 19.2%; dogs, 17.3%), independent of treatment. Although the rate of overall mortality was higher for AKI due to non-infectious etiologies for cats, the difference between non-infectious (53.3%) and infectious (30.0%) etiology groups was not statistically significant (Fig 2).

S6 Fig presents the results of a funnel plot of studies regarding overall mortality according to etiology for cats and dogs, suggesting some degree of publication bias with a potential relationship between treatment effect and study size.

#### Overall mortality according to dialysis treatment and AKI etiology

The pooled proportions for overall mortality according to dialysis treatment and AKI etiology were: AKI due to infectious etiology from eight case series studies [5–7,17,19,22,25,26] with a total of 156 cats and dogs, 22.0% [95% CI 0.14, 0.30; I^2^ = 23.5%, P = 0.242]; AKI due to non-infectious etiology from nine case series studies [5,7,12,17,19,21,25,26,28] with a total of 564 cats and dogs, 61.8% [95% CI 0.538, 0.696; I^2^ = 64.3%, P = 0.0042]; AKI due to infectious etiology from five cases series studies [5–7,25,26] with a total of 22 only cats, 30.0% [95% CI 0.13, 0.51; I^2^ = 21.5%, P = 0.2773]; AKI due to non-infectious etiology from seven case series studies [5–7,12,25,26,28] with a total of 309 only cats, 53.7% [95% CI 0.473, 0.601; I^2^ = 12.6%, P = 0.3336]; AKI due to infectious etiology from three cases series studies [17,19,22] with a total of 113 only dogs, 22.0% [95% CI 0.13, 0.32; I^2^ = 34.3%, P = 0.2182]; and AKI due to non-infectious etiology from four case series studies [7,17,19,21] with a total of 266 only dogs, 70.0% [95% CI 0.64, 0.75; I^2^ = 0%, P = 0.4936](S7 Fig). There was no significance heterogeneity across analyses, except with dialysis for cats and dogs with AKI due non-infectious etiology (P = 0.0042).

**Fig 3:**
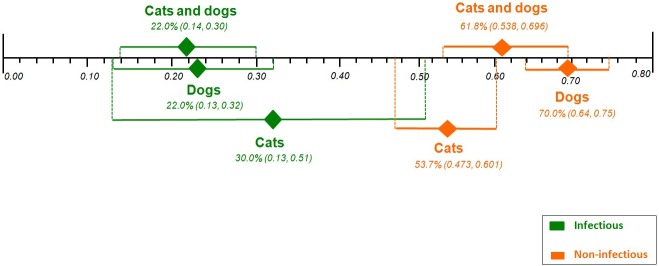
Comparison of the plotted proportional meta-analysis, according to dialysis treatment and etiology in both cats and dogs, for overall mortality. Effect differences were seen due to the non-overlap of the 95% confidence intervals showing a higher rates of mortality in the AKI due to non-infectious receiving dialysis (cats and dogs; dogs) compared with AKI due to infectious receiving dialysis (cats and dogs; dogs), as their CIs did not overlap. However, there was no statistically significance difference between rates of mortality in only cats that receiving dialysis, as their CIs overlapped.

Effect differences were seen, showing higher rates of mortality in AKI due to non-infectious etiology receiving dialysis (cats and dogs, 61.8% and; dogs, 70.0%) compared to those receiving dialysis with AKI due to infectious etiology (cats and dogs, 22.0% and; dogs, 22.0%). Although higher in the former group, there was no statistically significance difference in overall mortality between AKI due non-infectious etiology receiving dialysis (53.7%) and AKI due to infectious etiology receiving dialysis (30.0%) in cats (Fig 3).

#### Overall mortality according to control group treatment and AKI etiology

The pooled proportions for overall mortality according to control group treatment and AKI etiology were: AKI due to non-infectious etiology from two case series studies [23,28] with a total of 25 cats and dogs, 49.8% [95% CI 0.269, 0.727; I^2^ = not applicable, P = 0.2589]; AKI due to infectious etiology from three cases series studies [14,22,27] with a total of 168 only dogs, 14.0% [95% CI 0.08, 0.21; I^2^ = 15.4%, P = 0.3067] (S8 Fig). There was no significant heterogeneity across all analyses.

**Fig 4:**
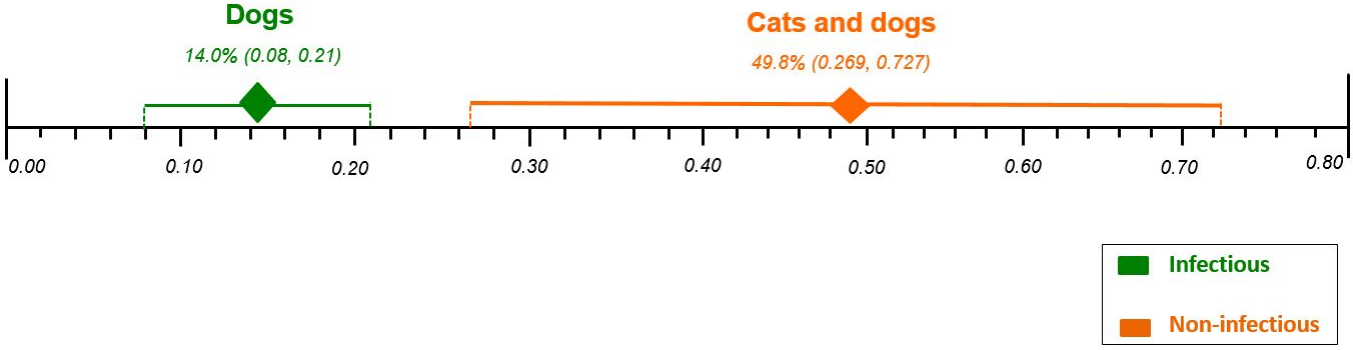
Comparison of the plotted proportional meta-analysis for overall mortality, according to control group treatment and AKI etiology in both cats and dogs. Effect differences were seen due to the non-overlap of the 95% CIs showing higher rates of mortality in cats and dogs with AKI due to non-infectious compared to only dogs with AKI due to infectious.

Effect differences were found, showing higher rates of mortality in cats and dogs with AKI due to non-infectious etiology (49.8%) compared to only dogs with AKI due to infectious etiology (14.0%). There were no enough studies to perform the proportional meta-analysis for the following: i) “only cats” and “only dogs” with AKI due to non-infectious etiology; ii) “cats and dogs” with AKI due to infectious etiology; and iii) “only cats” with AKI due to infectious etiology (Fig 4).

#### Euthanasia according to animal species

The pooled proportions for overall euthanasia according to animal species were: euthanasia from nine case series studies [5–7,12,13,24–26,28]with a total of 371only cats, 30.9% [95% CI 0.237, 0.386; I^2^ = 44.4%, P = 0.0724]; and euthanasia from 11 case series studies [1,7,13,14,19–24,27] with a total of 646 dogs, 28.79% [95% CI 0.1589, 0.4375; I^2^ = 92.8%, P < 0.0001] (S9 Fig). Significant heterogeneity was found in the analysis for euthanasia for dogs.

While the rate of overall euthanasia was higher for cats, independent of treatment and AKI etiology, no statistically-significant differences were found (S9 Fig).

S10 Fig presents the results of a funnel plot of studies regarding euthanasia for dogs by Egger's test. There is an asymmetrical that indicates a relationship between treatment effect and study size. This suggests the possibility of publication bias.

#### Euthanasia according to treatment

The pooled proportions for euthanasia according to treatment, independent of AKI etiology, were: dialysis treatment from 11 case series studies [5–7,12,13,19,21,22,25,26,28]with a total of 645cats and dogs, 28.7% [95% CI 0.223, 0.354; I^2^ = 57.3%, P = 0.0093]; control group from seven case series studies [14,20,22–24,27,28] with a total of 362 cats and dogs, 21.98% [95% CI 0.0585, 0.4463; I^2^ = 94.7%, P < 0.0001]; dialysis treatment from eight case series studies [5–7,12,13,25,26,28] with a total of 333 only cats, 29.0% [95% CI 0.22, 0.36; I^2^ = 33.3%, P = 0.1622]; dialysis treatment from five case series studies [7,13,19, 21,22] with a total of 312 only dogs, 30.8% [95% CI 0.193, 0.438; I^2^ = 71.2%, P = 0.0076]; control group from five case series studies [14,20,22,23,27] with a total of 296 only dogs, 16.17% [95% CI 0.0201, 0.4007; I^2^ = 94.7%, P < 0.0001]. (S11 Fig). Significant heterogeneity was found in all analyses, except for by dialysis (P = 0.1622). There were not enough studies to perform the proportional meta-analysis for the control groups from cats. (S11 Fig).

Although the rate of euthanasia was higher in the dialysis group, independent of AKI etiology and type of animals, there was no statistically significant difference among all studied groups (S11 Fig).

S12 Fig presents the results of a funnel plot of studies regarding euthanasia for cats and dogs that received dialysis, and suggests potential publication bias with a relationship between treatment effect and study size.

#### Complications according to animal species

The pooled proportions for overall complications according to animal species were: complications from two case series studies [6,26] with a total of 21only cats, 76.0% [95% CI 0.07, 0.93; I^2^ = not applicable, P = 0.0004]; complications from four case series studies [1,19,22,27] with a total of 360 dogs, 19.83% [95% CI 0.0025, 0.6832; I^2^ = 98.7, P < 0.0001] (S9 Fig). There was significant heterogeneity in all analyses.

While the rate of overall complications was higher for cats, regardless of treatment and AKI etiology, there were no statistically significant differences across all studied groups(S9 Fig).

#### Complications according to treatment

The pooled proportions for overall complications according to treatment, independent of AKI etiology, were: dialysis from four case series studies [6,19,22,26] with a total of 217 cats and dogs, 59.0% [95% CI 0.24, 0.89; I^2^ = 91.5%, P < 0.0001]; control group from two case series studies [22,27] with a total of 154 cats and dogs, 5.52% [95% CI 0.0025, 0.2509; I^2^ = not applicable, P = 0.009]; dialysis from two case series studies [6,26] with a total of 21 only cats, 76.0% [95% CI 0.07, 0.93; I^2^ = not applicable, P = 0.0004]; dialysis from two case series studies [19,22] with a total of 196 only dogs, 42.0% [95% CI 0.03, 0.89; I^2^ = not applicable, P < 0.0001]; control group from two case series studies [22,27] with a total of 154 dogs, 5.52% [95% CI 0.0025, 0.2509; I^2^ = not applicable, P = 0.009] (S11 Fig). There was significant heterogeneity in all analyses.

While the rate of complications was higher in the dialysis group, there was no significant difference among all studied groups. There were not enough studies to perform the proportional meta-analysis for the control groups from cats. (S11 Fig).

#### Non-resolution according to animal species

The pooled proportions for overall non-resolution according to animal species, independent of AKI etiology, were: non-resolution from five case series studies [5,6,25,26,28] with a total of 278 only cats, 6.6% [95% CI 0.018, 0.139; I^2^ = 62.3%, P = 0.0312]; and non-resolution from three case series studies [13,14,20] with a total of 133only dogs, 14.0% [95% CI 0.03, 0.31; I^2^ = 74.9%, P = 0.0185] (S9 Fig). There was significant heterogeneity in all analysis.

While the rate of overall non-resolution was higher for dogs, there were no statistically significant differences among all studied groups (S9 Fig).

#### Non-resolution according to treatment

The pooled proportions for overall non-resolution according to treatment were: dialysis from six case series studies [5,6,13,25,26,28] with a total of 296cats and dogs, 4.4% [95% CI 0.024, 0.071; I^2^ = not applicable, P = 0.4444]; control group from three case series studies [14,20,28] with a total of 117 cats and dogs, 24.0% [95% CI 0.02, 0.58; I^2^ = 84.4%, P = 0.0014]; dialysis from five case series studies [5,6,25,26,28] with a total of 274only cats, 3.8% [95% CI 0.019, 0.064; I^2^ = not applicable, P = 0.866]; and control group from two case series studies[14,20] with a total of 113 only dogs, 11.3% [95% CI 0.001, 0.435; I^2^ = not applicable, P = 0.0052]. (S11 Fig). There was significant heterogeneity in all analyses, except for dialysis for both cats and dogs, and for cats only.

Although the rate of non-resolution was higher in the control group, there were no statistically significant differences among all studied groups. There were not enough studies to perform the proportional meta-analysis for dialysis for dogs and control group for cats (S11 Fig).

#### Overall mortality, euthanasia, complications and non-resolution for cats and dogs

The pooled proportions for overall mortality, euthanasia, complications and non-resolution according to animal species, independent of AKI etiology and treatment, were: mortality from 17 case series studies [1,5–7,12–14,17,19–26,28] with a total of 1201 only cats and dogs, 47.2% [95% CI 0.382, 0.562; I^2^ = 88.3%, P < 0.0001]; mortality after sensitivity analysis without Francey 2002 from 17case series studies [1,5–7,12–14,19–28]with a total of 1077cats and dogs, 46.3% [95% CI 0.368, 0.560; I^2^ = 88.3%, P < 0.0001]; euthanasia from 17 case series studies [1,5–7,12–14,19–28] with a total of 1017 cats and dogs, 26.99% [95% CI 0.1814, 0.3687; I^2^ = 90.0%, P < 0.0001]; complications from six case series studies [1,6,19,22,26,27] with a total of 381 cats and dogs, 36.78% [95% CI 0.048, 0.7797; I^2^ = 98.2%, P < 0.0001]; and non-resolution from eight case series studies [5,6,13,14,20,25,26,28]with a total of 413 cats and dogs, 10.0% [95% CI 0.034, 0.195; I^2^ = 83.0%, P < 0.0001]; (S13 Fig). There was significant heterogeneity in all analyses.

A plausible sensitivity analysis excluding the Francey 2002 study from the primary analysis of overall mortality, given lack of reported euthanasia data, yielded results that were consistent with the primary analysis.

S14 Fig presents the results of a funnel plot of studies regarding overall mortality and euthanasia for cats and dogs, and indicates a relationship between treatment effect and study size suggesting possible publication bias.

## Discussion

### Main findings

#### Mortality

Our findings suggest that dogs and cats with AKI due to a non-infectious etiology have higher mortality rates than those with AKI due to infectious etiologies, regardless of the treatment received (Fig 2). Negative outcomes for dogs and cats with AKI of non-infectious etiology are likely largely attributed to nephrotoxic agents, of which ethylene glycol, with a mortality rate close to 100%, is most significant [21].

The combined mortality rate for dogs and cats with AKI is 47.2% (S13 Fig). Regardless of AKI etiology and treatment, cats were found to have non-significantly higher AKI-associated mortality rates (53.1%) than dogs (45%) (S1 Fig). A non-significantly elevated rate of mortality for AKI of non-infectious etiology compared to infectious etiology was found for cats (S5 Fig). We believe the lower rate of mortality found in dogs with AKI of infectious etiology may be a reflection of the low mortality risk associated with pyometra-associated AKI, where the prognosis is very favorable [27]. In contrast, drug-related nephrotoxicity appears to be more significant for cats. Further studies would be required to further elucidate the associated AKI-associated mortality risks associated with these specific etiologies for dogs and cats.

Our findings also suggest that regardless of etiology, dialysis treatment is associated with higher mortality rates compared to conservative treatment among cats and dogs, though the association is not statistically significant (S3 Fig). One must consider that the results may be potentially influenced by heterogeneity between studies, and by the increased severity of AKI for animals on dialysis relative to those being managed conservatively (88.4% vs. 11.6% rates of oliguria; see Table 1).

The inability to evaluate patients with AKI of the same severity comparing dialysis and conservative treatment makes it challenging to statistically prove that dialysis treatment is superior to conservative management in terms of overall mortality. However, many previous studies have shown the superiority of dialysis in the management of dogs and cats with severe AKI. It should be considered that the majority of these studies did not establish a rule or protocol to define when patients should undergo dialysis, which likely increases the heterogeneity between studies.

#### Complications and non-resolutions

The complication rate for cats and dogs with AKI is 36.78% (S13 Fig). The rate of complications was higher across all comparisons among animals treated with dialysis compared to those managed conservatively(S11 Fig). The higher rate of complications with dialysis may be explained by the need for catheter implantation and the associated risk of peritonitis, extravasation of fluid from the abdominal cavity, catheter obstruction and retention of dialysate (in cases of peritoneal dialysis). Other potential factors, which may explain this difference, include the risks of hypotension, dialysis imbalance syndrome, and hemodialysis thromboembolism, which are inherent to the procedure. Cats with AKI had a non-significantly higher complication rate compared to dogs (S9 Fig). This difference likely reflects the higher proportion of cats on dialysis when compared to dogs in the included studies (see Table 1). The non-resolution rate for dogs and cats with AKI is 10.0%(S13 Fig), with dogs having an elevated risk compared to cats (S9 Fig) and animals being managed conservatively having a higher risk than those receiving dialysis (S11 Fig).

#### Mortality and euthanasia

While dogs and cats with AKI were found to have a combined mortality rate of 46.3%, the rate of euthanasia in the same population is 27.0%. This suggests that over half of the dogs and cats with AKI who had fatal outcomes did so due to euthanasia (S13 Fig). While an animal’s health may be a primary consideration, euthanasia may also be motivated by an owner's financial inability to pay for treatment. Furthermore, higher rates of euthanasia were observed among animals receiving dialysis relative to those being managed conservatively. One must consider that these animals also may already have a lower probability of survival due to increased severity of their renal disease. As such, the appropriate management of AKI in cats and dogs may be influenced as much by appropriate measures to minimize exposure to contributory etiologies, and by effect, AKI severity, as it may be by appropriate treatment, access to healthcare and mitigation of financial barriers.

### Strengths and limitations

Strengths of our review include a comprehensive search strategy across multiple electronic databases, and a rigorous approach to screening, data abstraction and risk of bias assessment with paired and independent review.

The primary limitation of our review is significant heterogeneity between studies, given the included studies are case series which are retrospective in nature with small sample sizes and lacking control arms. Publication bias may also limit a number of the comparisons in the review. In addition, the evaluation of AKI non-resolution was limited to a very small number of studies. These limitations further compromise the generalizability of our review findings.

### Relation of prior work

A 2012reviewreportedthatmortality rates for cats with AKI of47-6%, even with dialysis and other treatments [29]. The review reported that worst prognosis was associated with oliguric and anuric animals compared to non-oliguric animals.

Segev and colleagues created a clinical score system to predict the prognosis of dogs and cats with AKI managed with hemodialysis [5,19]. The authors noted that while the predictive model was designed for animals on hemodialysis, its applicability in animals with less severe AKI that did not require hemodialysis was difficult to evaluate.

These findings are in line with our findings of higher mortality rates among animals on dialysis, where a larger proportion of oliguric animals were found, suggesting more severe renal disease.

Given the differences in mortality outcomes between animals with different renal disease severities, our findings support the notion that a new clinical model may be helpful in addition to markers such as serum creatinine and presence of oliguria, in predicting prognostic course and need for dialysis. Further studies may help elucidate the optimal time to initiate dialysis therapy and minimize complications as well.

## Implications

Evidence suggests statistically significantly higher rates of mortality in cats and dogs with AKI due to non-infectious etiology compared to those with AKI due to infectious etiology. Furthermore, there were non-statistically significant differences in mortality rates between dialysis and conservative management. Notable rates of complications and non-resolution (36.7% and 10.0%, respectively) were found. Euthanasia rates were found to represent over half of AKI-associated fatal outcomes.

Our findings are in line with previous investigations suggesting increased renal disease severity is likely associated with need for dialysis and worse outcomes. Further investigations regarding optimal time to initiate dialysis and the development of clinical models to prognosticate the course of disease and guide optimal treatment initiation for less severe cases of AKI in cats and dogs is warranted.

## Supporting information

S1 Table Searchstrategy. (TIF)

S1 Fig Overall mortality according to animal species. Panel A: Cats. Panel B: Dogs.
(TIF)

S2 Fig Funnel plot for the overall mortality for dogs. (TIF)

S3 Fig Overall mortality according to treatment. Panel A. Dialysis for both cats and dogs. Panel B. Control group for both cats and dogs. Panel C. Dialysis for cats. Panel D: Control group for cats. Panel E: Dialysis for dogs. Panel F: Control group for dogs.
(TIF)

S4 Fig Funnel plot for the overall mortality for cats and dogs that receiving dialysis. (TIF)

S5 Fig Overall mortality according to the etiology. Panel A: Infectious for cats and dogs. Panel B: Non-infectious for cats and dogs. Panel C: Infectious for cats. Panel D: Non-Infectious for cats. Panel E: Infectious for dogs. Panel F: Non-infectious for dogs.
(TIF)

S6 Fig Funnel plot for the overall mortality according to etiology. Panel A: Infectious for cats and dogs. Panel B: Non-infectious for cats and dogs.
(TIF)

S7 Fig Overall mortality according to dialysis treatment, etiology in both cats and dogs. Panel A: Cats and dogs due infectious. Panel B: Cats and dogs due non-infectious. Panel C: Cats due infectious. Panel D: Cats due non-infectious. Panel E: Dogs due infectious. Panel F: Dogs due non-infectious.
(TIF)

S8 Fig Overall mortality according to control group treatment, etiology in both cats and dogs. Panel A: Cats and dogs due non-infectious. Panel B: Dogs due infectious.
(TIF)

S9 Fig Euthanasia, complications and non-resolution according to animal species. Panel A: Euthanasia from cats. Panel B: Euthanasia from dogs. Panel C: Complications from cats. Panel D: Complications from dogs. Panel E: Non-resolution from cats. Panel F: Non-resolution from dogs.
(TIF)

S10 Fig Funnel plot for the euthanasia for dogs. (TIF)

S11 Fig Euthanasia, complications and non-resolution according to treatment. Panel A: Euthanasia and dialysis for cats and dogs. Panel B: Euthanasia and control group for cats and dogs Panel C: Complications and dialysis for cats and dogs. Panel D: Complications and control group for cats and dogs. Panel E: Non-resolution and dialysis for cats and dogs. Panel F: Non-resolution and control group for cats and dogs. Panel G: Euthanasia and dialysis for cats. Panel H: Complications and dialysis for cats. Panel I: Non-resolution and dialysis for cats. Panel J: Euthanasia and dialysis for dogs. Panel K: Euthanasia and control group for dogs. Panel L: Complications and dialysis for dogs Panel M: Complications and control group for dogs. Panel N: Non-resolution and control group for dogs.
(TIF)

S12 Fig Funnel plot for the euthanasia for cats and dogs that receiving dialysis. (TIF)

S13 Fig Overall mortality, euthanasia, complications and non-resolution for cats and dogs. Panel A: Mortality for cats and dogs. Panel B: Sensitivity analysis without Francey 2002, for overall mortality. Panel C: Euthanasia for cats and dogs. Panel D: Complications for cats and dogs. Panel E: Non-resolution for cats and dogs.
(TIF)

S14 Fig Funnel plot for the overall mortality and euthanasia for cats and dogs. Panel A: Mortality for cats and dogs. Panel B: Sensitivity analysis without Francey 2002, for overall mortality. Panel C: Euthanasia for cats and dogs.
(TIF)
