# Supplementary figures and images for: Acute kidney injury in cats and dogs: A proportional meta-analysis of case series studies
Source: PLoS One. 2018 Jan 25;13(1):e0190772. doi: 10.1371/journal.pone.0190772 (PMC5784898; doi:10.1371/journal.pone.0190772)

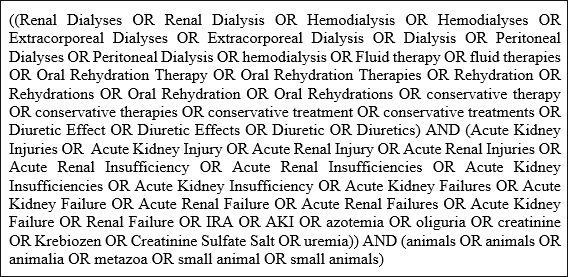

Supplement: S1 Table — (TIF) [file pone.0190772.s001.tif]

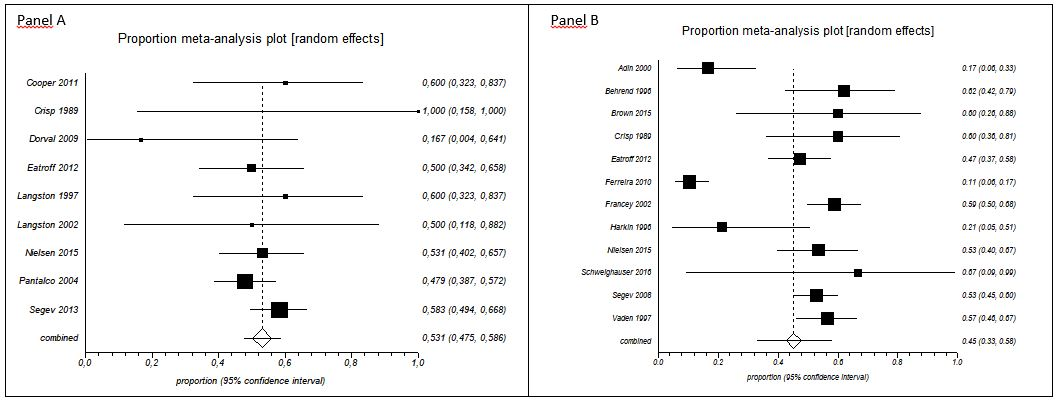

Supplement: S1 Fig — Panel A: Cats. Panel B: Dogs. (TIF) [file pone.0190772.s002.tif]

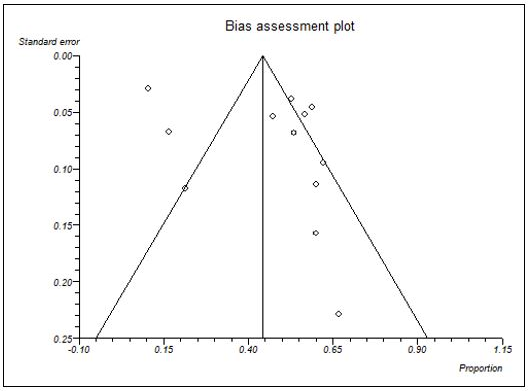

Supplement: S2 Fig — (TIF) [file pone.0190772.s003.tif]

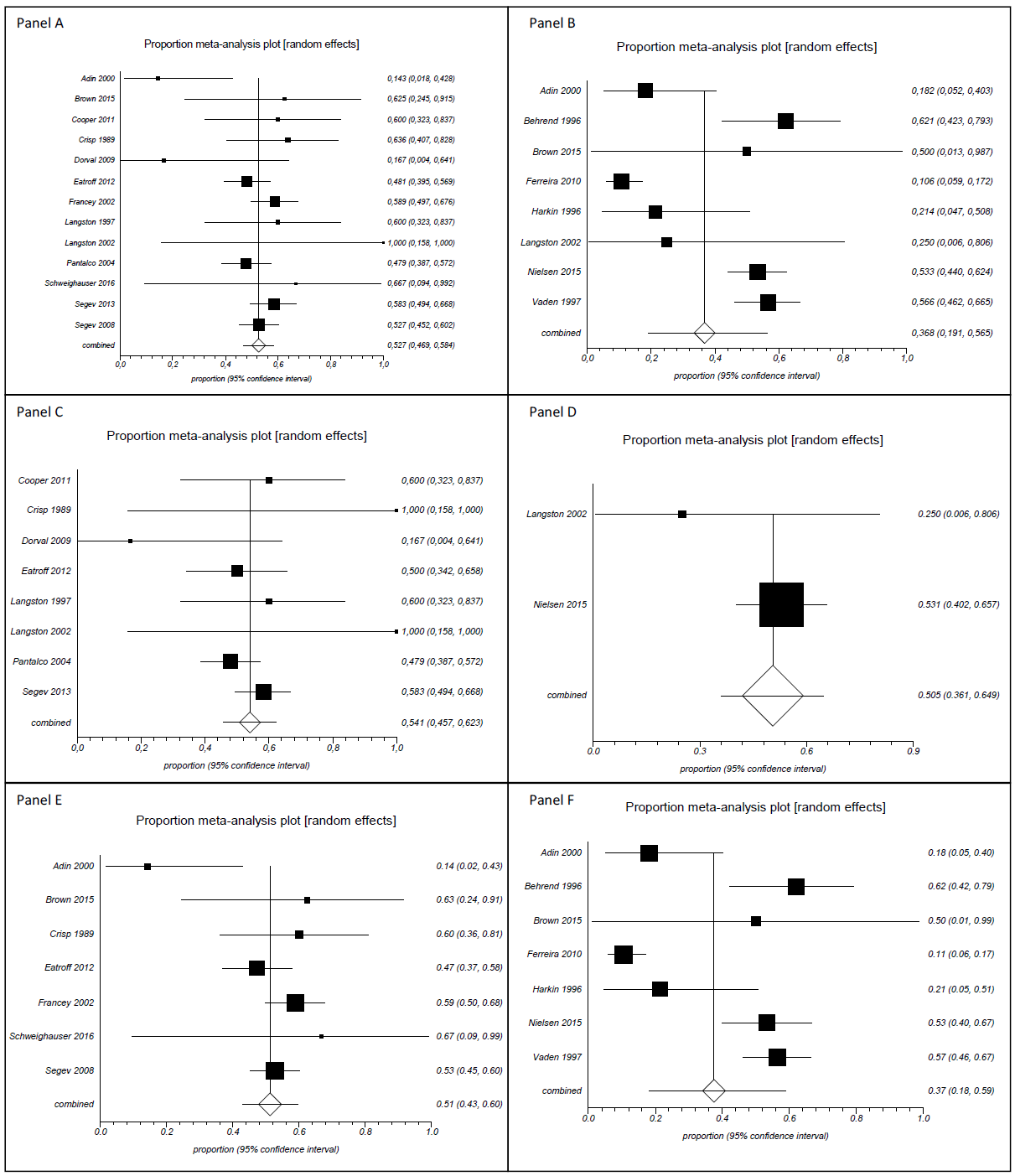

Supplement: S3 Fig — Panel A. Dialysis for both cats and dogs. Panel B. Control group for both cats and dogs. Panel C. Dialysis for cats. Panel D: Control group for cats. Panel E: Dialysis for dogs. Panel F: Control group for dogs. (TIF) [file pone.0190772.s004.tif]

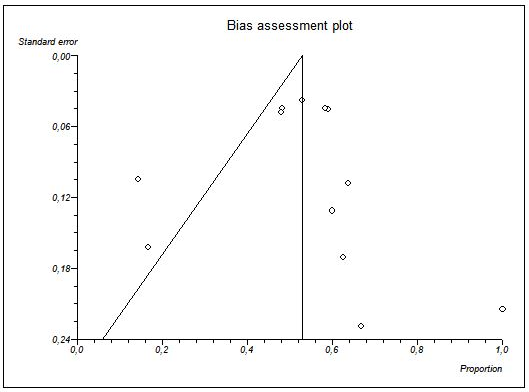

Supplement: S4 Fig — (TIF) [file pone.0190772.s005.tif]

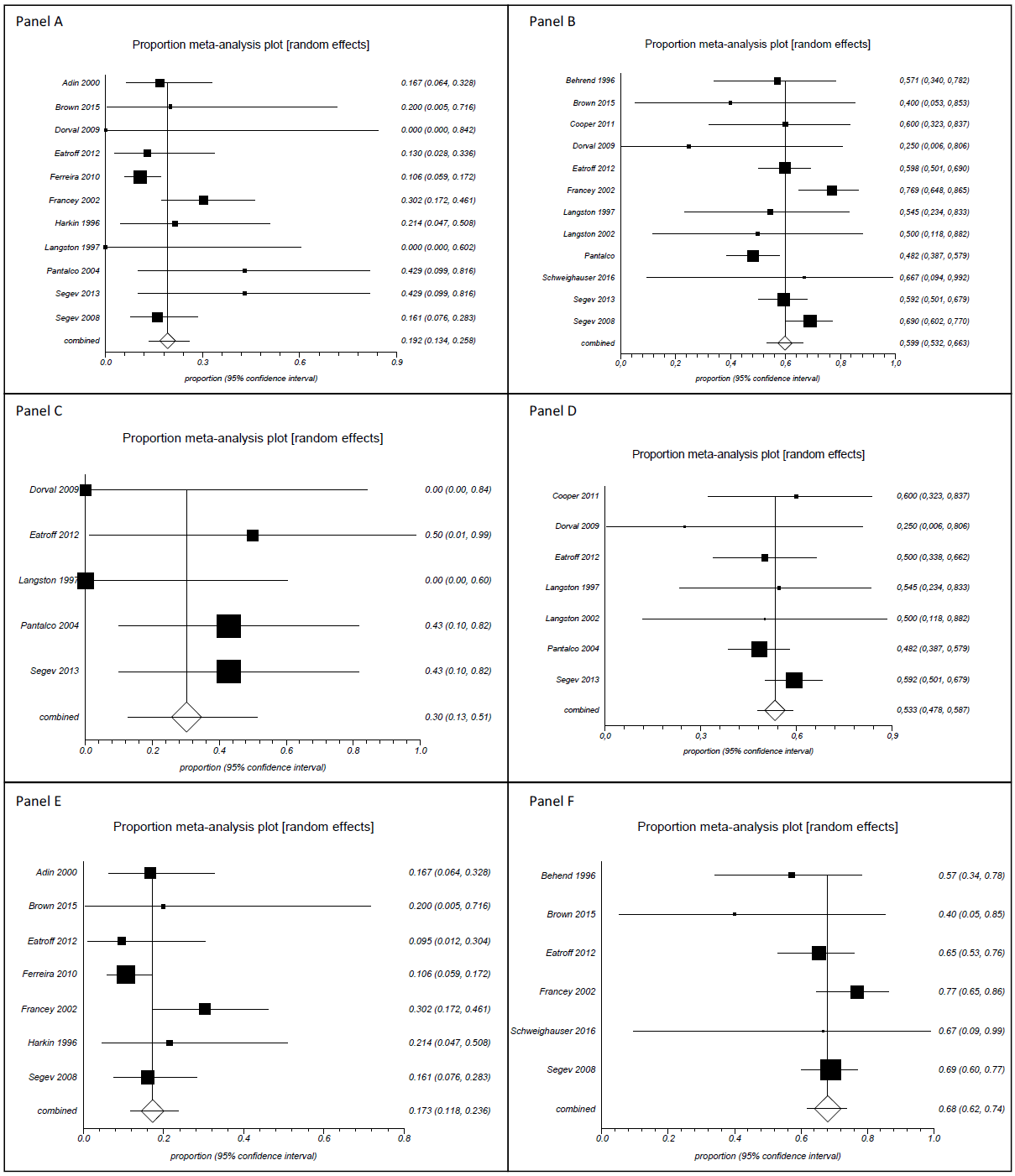

Supplement: S5 Fig — Panel A: Infectious for cats and dogs. Panel B: Non-infectious for cats and dogs. Panel C: Infectious for cats. Panel D: Non-Infectious for cats. Panel E: Infectious for dogs. Panel F: Non-infectious for dogs. (TIF) [file pone.0190772.s006.tif]

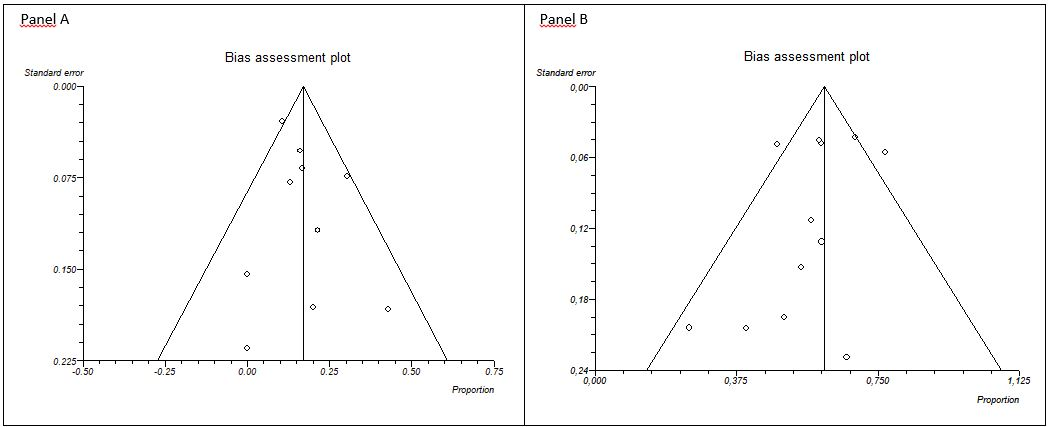

Supplement: S6 Fig — Panel A: Infectious for cats and dogs. Panel B: Non-infectious for cats and dogs. (TIF) [file pone.0190772.s007.tif]

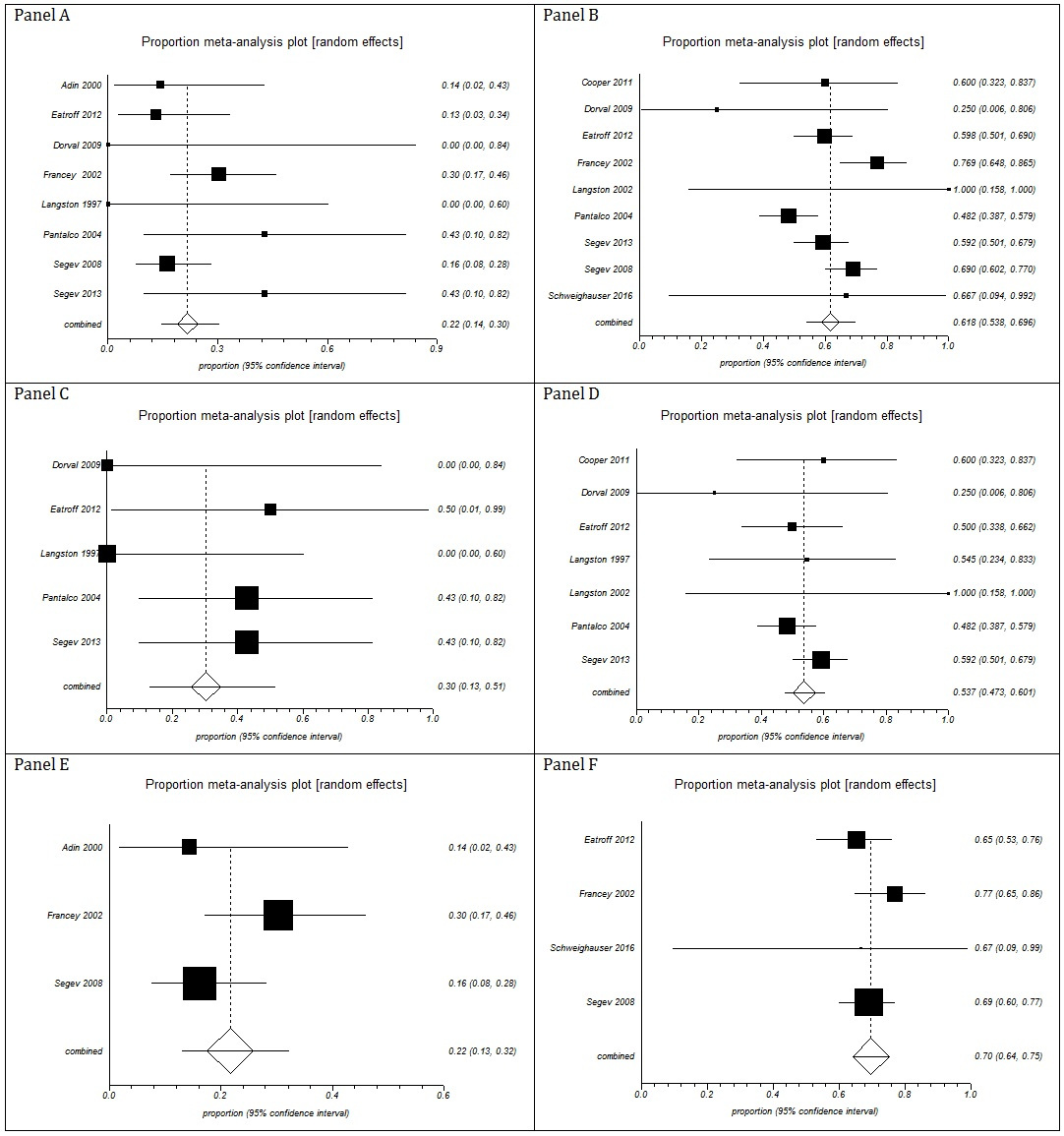

Supplement: S7 Fig — Panel A: Cats and dogs due infectious. Panel B: Cats and dogs due non-infectious. Panel C: Cats due infectious. Panel D: Cats due non-infectious. Panel E: Dogs due infectious. Panel F: Dogs due non-infectious. (TIF) [file pone.0190772.s008.tif]

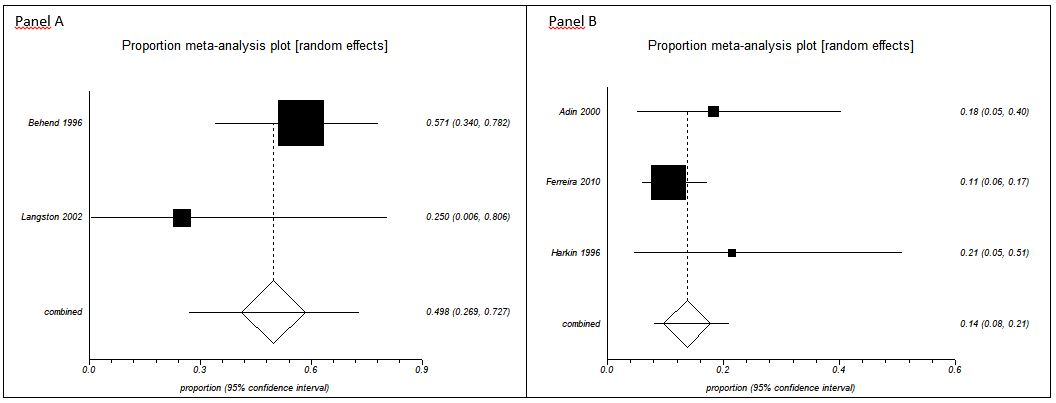

Supplement: S8 Fig — Panel A: Cats and dogs due non-infectious. Panel B: Dogs due infectious. (TIF) [file pone.0190772.s009.tif]

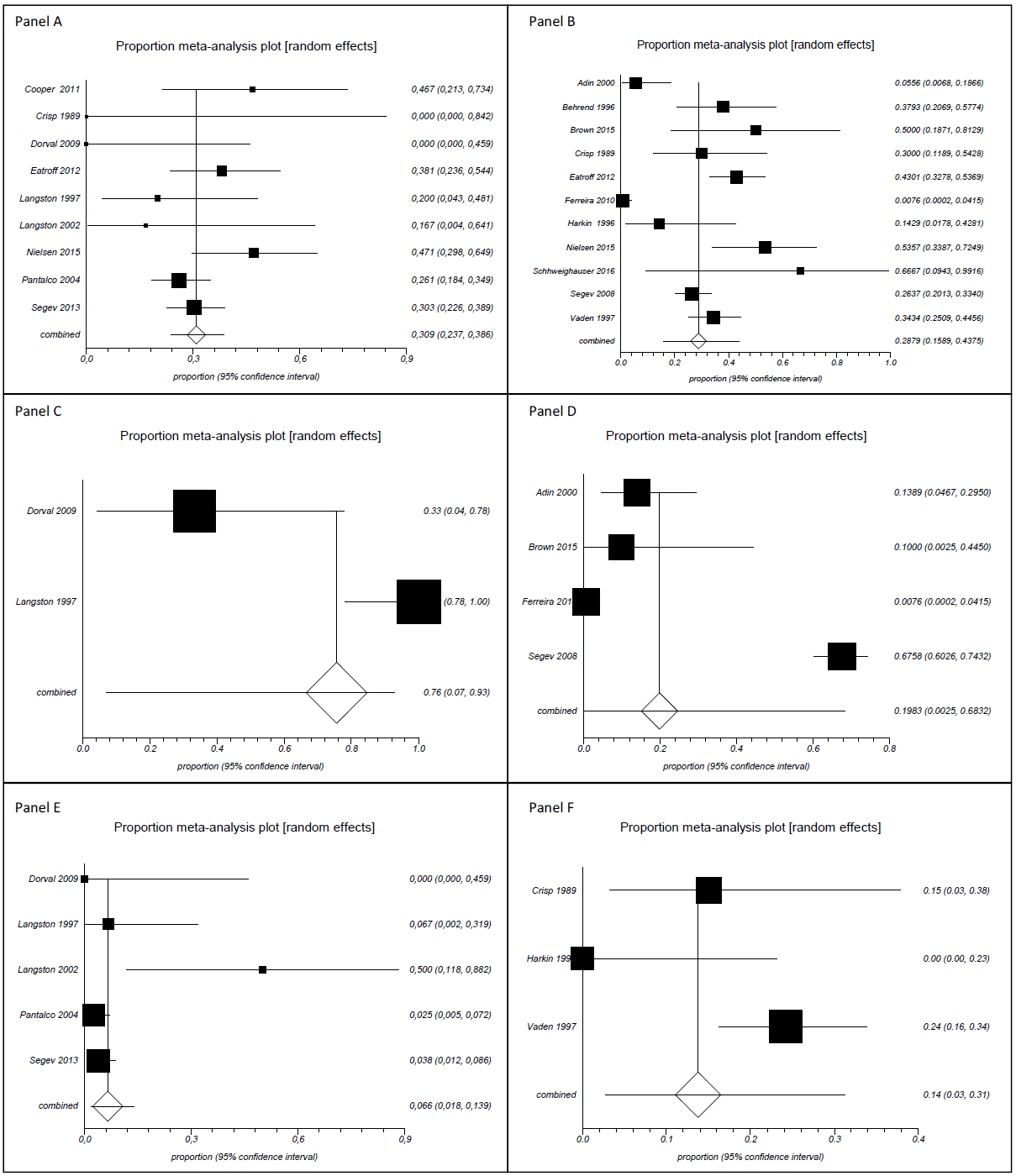

Supplement: S9 Fig — Panel A: Euthanasia from cats. Panel B: Euthanasia from dogs. Panel C: Complications from cats. Panel D: Complications from dogs. Panel E: Non-resolution from cats. Panel F: Non-resolution from dogs. (TIF) [file pone.0190772.s010.tif]

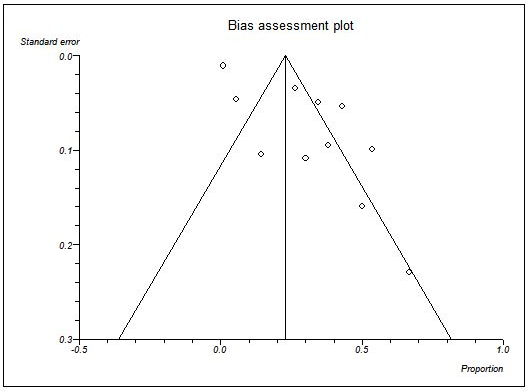

Supplement: S10 Fig — (TIF) [file pone.0190772.s011.tif]

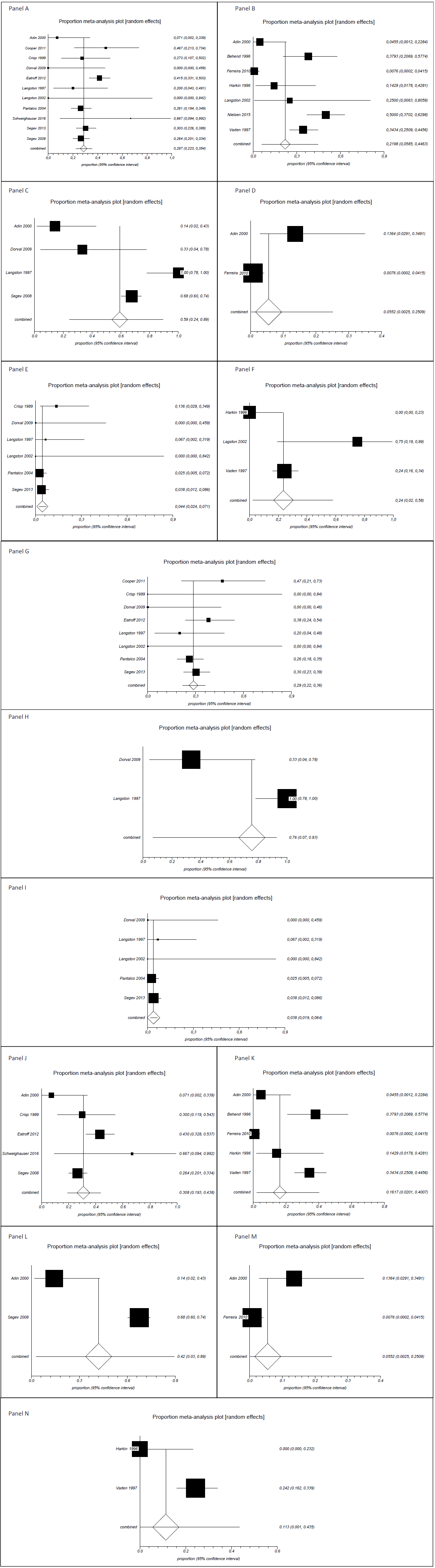

Supplement: S11 Fig — Panel A: Euthanasia and dialysis for cats and dogs. Panel B: Euthanasia and control group for cats and dogs Panel C: Complications and dialysis for cats and dogs. Panel D: Complications and control group for cats and dogs. Panel E: Non-resolution and dialysis for cats and dogs. Panel F: Non-resolution and control group for cats and dogs. Panel G: Euthanasia and dialysis for cats. Panel H: Complications and dialysis for cats. Panel I: Non-resolution and dialysis for cats. Panel J: Euthanasia and dialysis for dogs. Panel K: Euthanasia and control group for dogs. Panel L: Complications and dialysis for dogs Panel M: Complications and control group for dogs. Panel N: Non-resolution and control group for dogs. (TIF) [file pone.0190772.s012.tif]

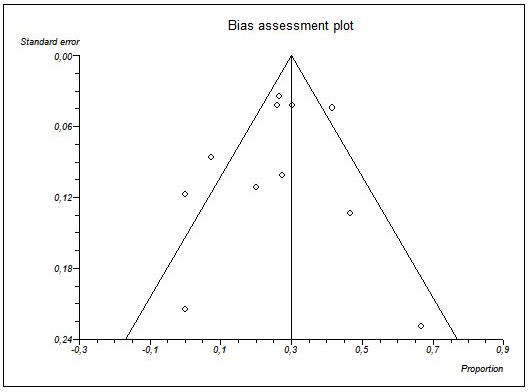

Supplement: S12 Fig — (TIF) [file pone.0190772.s013.tif]

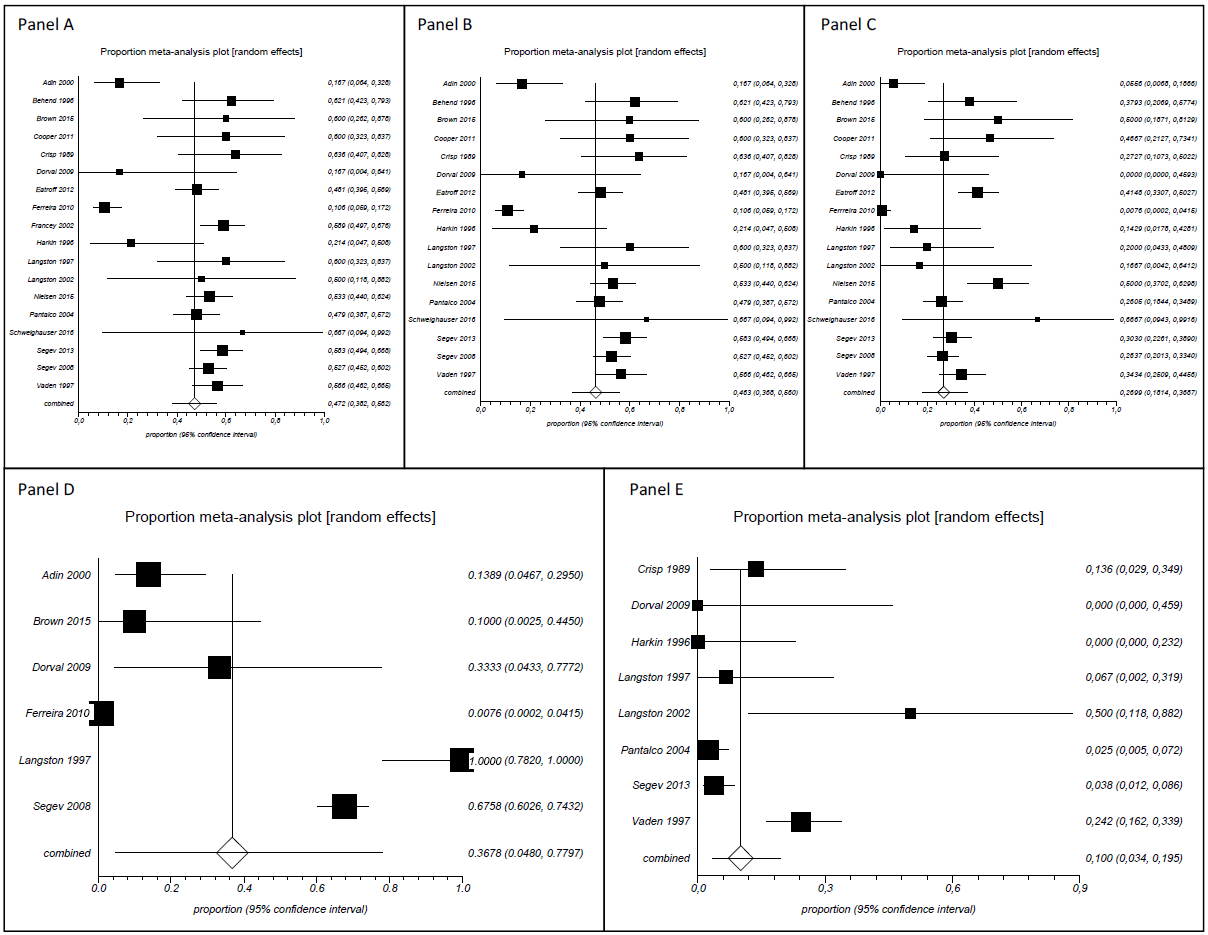

Supplement: S13 Fig — Panel A: Mortality for cats and dogs. Panel B: Sensitivity analysis without Francey 2002, for overall mortality. Panel C: Euthanasia for cats and dogs. Panel D: Complications for cats and dogs. Panel E: Non-resolution for cats and dogs. (TIF) [file pone.0190772.s014.tif]

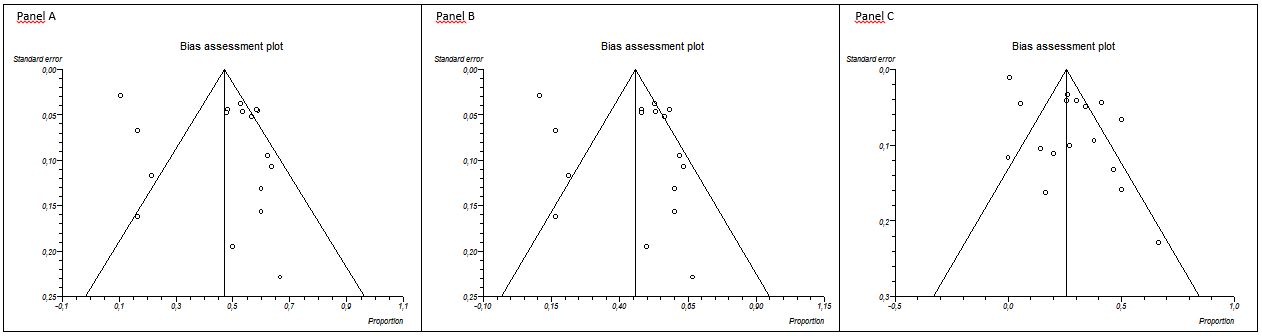

Supplement: S14 Fig — Panel A: Mortality for cats and dogs. Panel B: Sensitivity analysis without Francey 2002, for overall mortality. Panel C: Euthanasia for cats and dogs. (TIF) [file pone.0190772.s015.tif]
